# Curated MicroRNAs in Urine and Blood Fail to Validate as Predictive Biomarkers for High-Risk Prostate Cancer

**DOI:** 10.1371/journal.pone.0091729

**Published:** 2014-04-04

**Authors:** Nikhil Sapre, Matthew K. H. Hong, Geoff Macintyre, Heather Lewis, Adam Kowalczyk, Anthony J. Costello, Niall M. Corcoran, Christopher M. Hovens

**Affiliations:** 1 Division of Urology, Department of Surgery, Royal Melbourne Hospital and the University of Melbourne, Parkville, Victoria, Australia; 2 Australian Prostate Cancer Research Epworth, Richmond, Victoria, Australia; 3 NICTA Victoria Research Laboratory, Department of Electronic and Electrical Engineering, The University of Melbourne, Parkville, Victoria, Australia; 4 Department of Computing and Information Systems, The University of Melbourne, Parkville, Victoria, Australia; IPO, Inst Port Oncology, Portugal

## Abstract

**Purpose:**

The purpose of this study was to determine if microRNA profiling of urine and plasma at radical prostatectomy can distinguish potentially lethal from indolent prostate cancer.

**Materials and Methods:**

A panel of microRNAs was profiled in the plasma of 70 patients and the urine of 33 patients collected prior to radical prostatectomy. Expression of microRNAs was correlated to the clinical endpoints at a follow-up time of 3.9 years to identify microRNAs that may predict clinical response after radical prostatectomy. A machine learning approach was applied to test the predictive ability of all microRNAs profiled in urine, plasma, and a combination of both, and global performance assessed using the area under the receiver operator characteristic curve (AUC). Validation of urinary expression of miRNAs was performed on a further independent cohort of 36 patients.

**Results:**

The best predictor in plasma using eight miRs yielded only moderate predictive performance (AUC = 0.62). The best predictor of high-risk disease was achieved using miR-16, miR-21 and miR-222 measured in urine (AUC = 0.75). This combination of three microRNAs in urine was a better predictor of high-risk disease than any individual microRNA. Using a different methodology we found that this set of miRNAs was unable to predict high-volume, high-grade disease.

**Conclusions:**

Our initial findings suggested that plasma and urinary profiling of microRNAs at radical prostatectomy may allow prognostication of prostate cancer behaviour. However we found that the microRNA expression signature failed to validate in an independent cohort of patients using a different platform for PCR. This highlights the need for independent validation patient cohorts and suggests that urinary microRNA signatures at radical prostatectomy may not be a robust way to predict the course of clinical disease after definitive treatment for prostate cancer.

## Introduction

Prostate cancer is characterised by its distinctly variable and unpredictable outcomes. The introduction of prostate specific antigen (PSA) testing has resulted in a dramatic increase in early detection of prostate cancer (CaP) and a stage migration such that more men with early stage CaP are now being diagnosed with the disease [1], [2]. However, the test is neither specific for CaP [3] nor does it allow for accurate risk stratification and selection of those patients likely to succumb to their disease after early radical therapy [4], [5]. The morbidity associated with typical treatments has given rise to a plethora of biomarker development studies in an attempt to identify patients most likely to benefit from timely intervention to reduce their risk of recurrence and metastasis [6].

Although much research is aimed at finding biomarkers that can improve prostate cancer detection rates over PSA, the key issue clinically, is the detection of high-risk CaP at an early, curable stage. Whilst the majority of cases follow an indolent course and do not require curative treatment, some cancers have the potential to metastasize and require aggressive, early, clinical intervention. However current clinicopathological models do not allow clinicians to accurately discern at an early stage between high risk and indolent CaP [7]. There is an urgent clinical need for new markers that will discriminate indolent from aggressive prostate cancers at an early stage.

MicroRNAs (miRNAs) are approximately 22 nucleotide-long, single-stranded, non-coding RNAs that bind to complementary “seed” regions found in the 3′ untranslated region (UTR) of particular messenger RNA (mRNA) species. MiRNAs modulate expression of their mRNA targets, either marking them for destruction or inhibiting their binding to translational machinery [8]. MiRNAs have been shown to be involved in a wide range of important physiological and pathological processes including cell cycle processes, development, survival, differentiation, growth, apoptosis and immune response [8].

Several studies have shown the deregulation of miRNA is an important mechanism in prostate carcinogenesis [9] and that such changes can be detected in the sera of CaP patients [10], [11], [12], [13]. There are several advantages of miRNAs as biomarkers in biofluids. Biofluids such as plasma and urine are less invasive to obtain compared to tissue and miRNAs are frequently deregulated in cancer and exhibit tissue specific expression. In addition, their expression in blood and urine is stable and can be quantified sensitively by quantitative real time polymerase chain reaction (RT-PCR)[14]. There has thus been much excitement about the potential of miRNAs as biomarkers of CaP. However few of these studies have performed systematic validation of these biomarkers, which has prevented their clinical translation in management of CaP. Additionally, the majority of these studies that have compared early CaP to advanced/metastatic CaP have used samples collected once the patients have developed metastatic disease. The aim of this study was to investigate if the profiling of a panel of miRNAs in the plasma and urine of primary prostate cancer patients prior to radical prostatectomy can distinguish indolent CaP from the high-risk phenotype and to validate any findings in an independent cohort of patients.

## Materials and Methods

### Ethics Statement

This project had full ethics approval from institutional review boards. The ethics approvals pertaining to this manuscript are Royal Melbourne Hospital Human Research Ethics Committee (HREC) No 2006.073 and Epworth Healthcare HREC No. 34506. All patients gave full written consent for their samples to be stored and used for research using patient information and consent forms that were approved by the ethics committees.

### MicroRNA panel selection

A systematic literature review was conducted in PubMed to find microRNAs implicated in prostate and epithelial cancer initiation, progression and metastasis using the terms ‘prostate cancer’ and ‘microRNA’ in July 2011. The cited references of studies were checked for further articles of interest. All articles including original articles, reviews and abstracts were considered. Based on our review we chose a panel of 12 miRs for profiling in plasma and 13 miRs for profiling in urine, all of which had at least 2 independent studies published showing involvement in prostate cancer carcinogenesis, with preference given to those with concomitant supporting mechanistic data. In addition we included RNU48 in our panel as a putative endogenous control [15].

### Patient Selection

All patients with a diagnosis of CaP who underwent radical prostatectomy at the Epworth Hospital from 2003 to 2010 with detailed PSA follow-up and complete clinical and pathological data were identified from a prospectively recorded and maintained dedicated prostate cancer database. The first cohort consisted of patients with high-risk phenotype prostate cancer (n = 33 plasma samples, 16 urine samples) and patients with low-risk or indolent phenotype cancer (n = 37 plasma samples, 17 urine samples). The second (validation) cohort consisted of patients with high-risk phenotype prostate cancer (n = 22 urine samples) and patients with indolent phenotype prostate cancer (n = 14 urine samples). High-risk cancer was defined as Gleason grade greater than or equal to 7 and tumour volume >1cc or development of metastatic disease or early biochemical recurrence suggestive of systemic micro metastases (short PSA doubling time, failed salvage radiotherapy, PSA persistence). Indolent cancer was defined as Gleason 6, T2a, PSA<10 and PSA-free survival for at least 3 years (Epstein criteria)[16]. Plasma and urine were collected from patients prior to prostatectomy immediately after a digital rectal examination, snap-frozen and stored in liquid nitrogen.

### Clinical data collection

Relevant clinical and pathological data were recorded prospectively and analysed retrospectively. Details of PSA follow-up were recorded prospectively and updated annually. Tumour volumes were measured accurately by computed planimetry at the time of histological assessment as previously described [17]. The TNM (2002) classification system was used to stage the specimens. The Gleason sum score, and pathologic tumour stage were all assessed separately. For patients who did not experience a biochemical recurrence during the study period, follow-up was censored at the time of their last recorded PSA test. For patients with more than a single postoperative PSA recorded, PSA doubling time (PSAdt) was calculated using the log slope method [18]. For this study, biochemical recurrence was defined as a postoperative PSA value greater than or equal to 0.2 ng/ml and rising, or a rising PSA level below this threshold that was felt by the treating physician to represent a recurrence and led to the institution of salvage therapy. Significant biochemical recurrence was defined as PSA recurrence with a doubling time <6 months, as this has been shown to be a more accurate predictor of the development of metastases and cancer-specific mortality than PSA recurrence alone [19], [20]. For patients with primary PSA persistence who were immediately treated with salvage therapy the PSAdt was arbitrarily assumed to be <6 months. Collection and use of this information had Melbourne Health Ethics Committee approval.

### RNA extraction

500 µl of plasma or urine was thawed on ice for total RNA extraction using the mirVana miRNA Isolation Kit (Ambion, TX, USA) according to the manufacturer's protocol with the following modifications/conditions: 1.5 volumes of Lysis/Binding Buffer was used for cell lysis, before addition of homogenate, and RNA was eluted in 40 µl water. RNA elutions were frozen on dry ice and stored in −80°C.

### RT-PCR

RNA was concentrated from 40 µl to 15 µl using a Savant SpeedVac (Thermo Fisher Scientific, NC, USA) at 45°C/high pressure. Reverse transcription was performed on a thermal cycler (Applied Biosystems, CA, USA) using the Taqman microRNA Reverse Transcription Kit (Applied Biosystems, CA, USA) according to manufacturer's small RNA assay protocol with the following modifications: 7 µl RNA was used as template and pooled primers were used for the pre-selected 14 miRNA species.

#### Original Cohort

Quantitative real-time PCR was performed with the resulting cDNA on custom Taqman Low Density Array Cards (Applied Biosystems, CA, USA) containing primers for our miRNA panel according to manufacturer's protocol. All reactions were performed in triplicate and the median included in the final analysis. TLDA cards were run on a 7900HT Fast Real-Time PCR System.

#### Validation Cohort

Preamplification of the synthesised cDNA was performed according to the manufacturer's instructions. Briefly, 2.5 µl cDNA was added to 12.5 µl preamplification MasterMix, 3.75 ul primer pool and 6.25 µl nuclease-free water. This reaction was heated to 95°C for 10 min, 55°C for 2 min, 72°C for 2 min followed by 12 cycles of amplification before heating to 99°C for 10 min. The amplified reaction products were diluted to 200 µl using 0.1× Tris-EDTA buffer (pH = 8). RT-PCR was perfomed on the ViiA 7 (Applied Biosystems, CA, USA) using 0.5 ul 20× miRNA assay, 0.1 µl preamp product, 5.0 µl TaqMan Fast Universal PCR Master Mix (2⊥), 4.4 µl nuclease-free water were combined for a 10 ul PCR reaction. All reactions were performed in triplicate and the median included in the final analysis.

### Data analysis

#### Pre-processing

Thresholds for the PCR runs were set using RQ Manager (Applied Biosystems) and manually checked to ensure the cT corresponded to the midpoint of the logarithmic amplification. All observed Ct values greater than 34 were considered not expressed and set to 34. Any undetermined Ct values were also set to 34. As each miR was profiled in triplicate, any replicate value more than 20% different from the remaining two values was considered an outlier and removed from analysis. The mean Ct value was then determined for each sample and miR across replicates.

#### Normalisation

**Figure 1:**
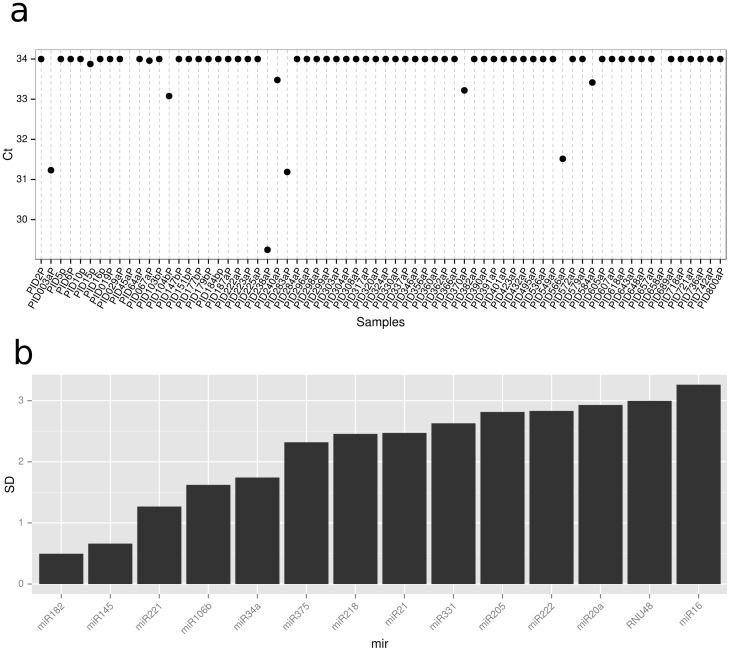
Plots for assessing endogenous controls. a) Ct values for the endogenous control across all samples in the plasma cohort. A Ct of 34 is considered undetected. b) the standard deviation of Ct values across samples for each miR in the original urine cohort.

RNU48 was profiled in each samples as an endogenous control but was deemed inappropriate for plasma as in the majority of samples it was not detected (Figure 1a), deemed inappropriate for urine as it was the miR with the third highest standard deviation (Figure 1b), and showed variable expression between high-risk and low-risk groups. Therefore, geometric mean normalisation was used, a normalisation shown to be effective in miR PCR profiling [21]

#### Differential expression analysis

The mean Ct of samples belonging to high-risk and low-risk groups was used to calculate ΔΔCt. Fold-change was calculated as 2^−ΔΔCt^. A student t-test was used to calculate the significance of the difference between high and low risk groups and the p-value was adjusted for multiple testing correction using the Benjamini-Hochberg method [22]

#### Feature selection and classifiaction

We assume the data is given as a matrix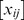, N features (miRs) for *M* samples i.e. 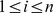 and 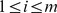 with the label vector 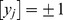 arranged in such a way that 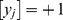
for 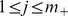 and 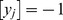 for the remaining samples 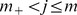. We have used two different methods for selection/ordering of features/miRs from most to the least “discriminating”. The first method used was the classical Student's t-test, which allocated to the *i*-th feature the score
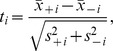
where
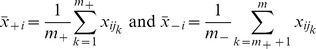
denote the means and
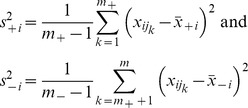
denote the variances of the *i*-th variable for samples of the both groups of interest, respectively. Then the features are ranked in the descending order of absolute values of the statistic 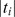, for 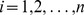. The second technique, named *the centroid feature selection*, ranks the feature in the descending order of the magnitude 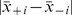 of the difference between means of the *i*-th variable for samples of the both groups of interest.

For various subsets of *t* top features, 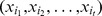, we generated linear classifiers
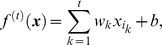
for a sample 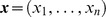, using a support vector machine algorithm, which selected weights *w_k_* and the intercept *b* by minimising the following functional:
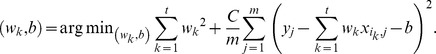
Here *C* is adjustable regularisation constant, with a relatively weak impact on the final result in our case. The results reported have used *C* = 1.

## Results

The demographics and clinicopathological characteristics of the low-risk and high-risk patients in cohort 1 (discovery cohort) in the plasma and urine profiling arms are outlined in Table 1.

**Table 1:** Clinical and pathological characteristics of the patient cohort in the original cohort.

		Plasma cohort		Urine cohort	
		Low-Risk	High-Risk	Low-Risk	High-Risk
n		33	37	17	16
Age	Median	64	67	63	66
(yrs)	Range	48–74	57–81	52–72	57–81
Follow-up	Median	3.89	4.44	4.24	4.39
(yrs)	Range	1.16–6.02	1.65–7.01	3.65–5.05	2.12–5.10
Prostate Weight	Median	58	46	58	48
(g)	Range	28–86	20–118	34–86	20–64
Gleason Grade (%)	6	33 (100)	0 (0)	17 (100)	0 (0)
	7 (3+4)	0 (0)	7 (19)	0 (0)	1 (6)
	7 (4+3)	0 (0)	8 (22)	0 (0)	3 (19)
	8	0 (0)	3 (8)	0 (0)	1 (6)
	9 (4+5)	0 (0)	16 (43)	0 (0)	8 (50)
	9 (5+4)	0 (0)	3 (8)	0 (0)	3 (19)
Tumour Volume	Median	0.20	7.76	0.20	9.45
(cc)	Range	0.04–0.90	0.85–28.70	0.09–0.80	1.40–25.20
pT stage (%)	pT2a	12 (36)	0 (0)	6 (35)	0 (0)
	pT2b	0 (0)	2 (5)	0 (0)	1 (6)
	pT2c	21 (64)	6 (16)	11 (65)	3 (19)
	pT3a	0 (0)	13 (35)	0 (0)	5 (31)
	pT3b	0 (0)	16 (44)	0 (0)	7 (44)
EPE (%)	Present	0 (0)	27 (73)	0 (0)	10 (63)
	Absent	33 (100)	10 (27)	17 (100)	6 (37)
SV Invasion (%)	Present	0 (0)	5 (14)	0 (0)	1 (6)
	Absent	33 (100)	32 (86)	17 (100)	15 (94)
PN Invasion (%)	Present	7 (21)	35 (95)	2 (12)	16 (100)
	Absent	26 (79)	2 (5)	15 (88)	0 (0)
LV Invasion (%)	Present	0 (0)	18 (49)	0 (0)	9 (56)
	Absent	33 (100)	19 (51)	17 (100)	7 (44)
Multifocal (%)	Yes	25 (76)	25 (68)	12 (71)	10 (63)
	No	8 (24)	12 (32)	5 (29)	6 (37)
Surgical Margins (%)	Positive	1 (3)	26 (70)	1 (6)	9 (56)
	Negative	32 (97)	11 (30)	16 (94)	7 (44)
Metastatic Disease (%)	Yes	0 (0)	5 (14)	0 (0)	2 (13)
	No	33 (100)	32 (86)	17 (100)	14 (87)

### microRNA profiling of plasma

**Figure 2:**
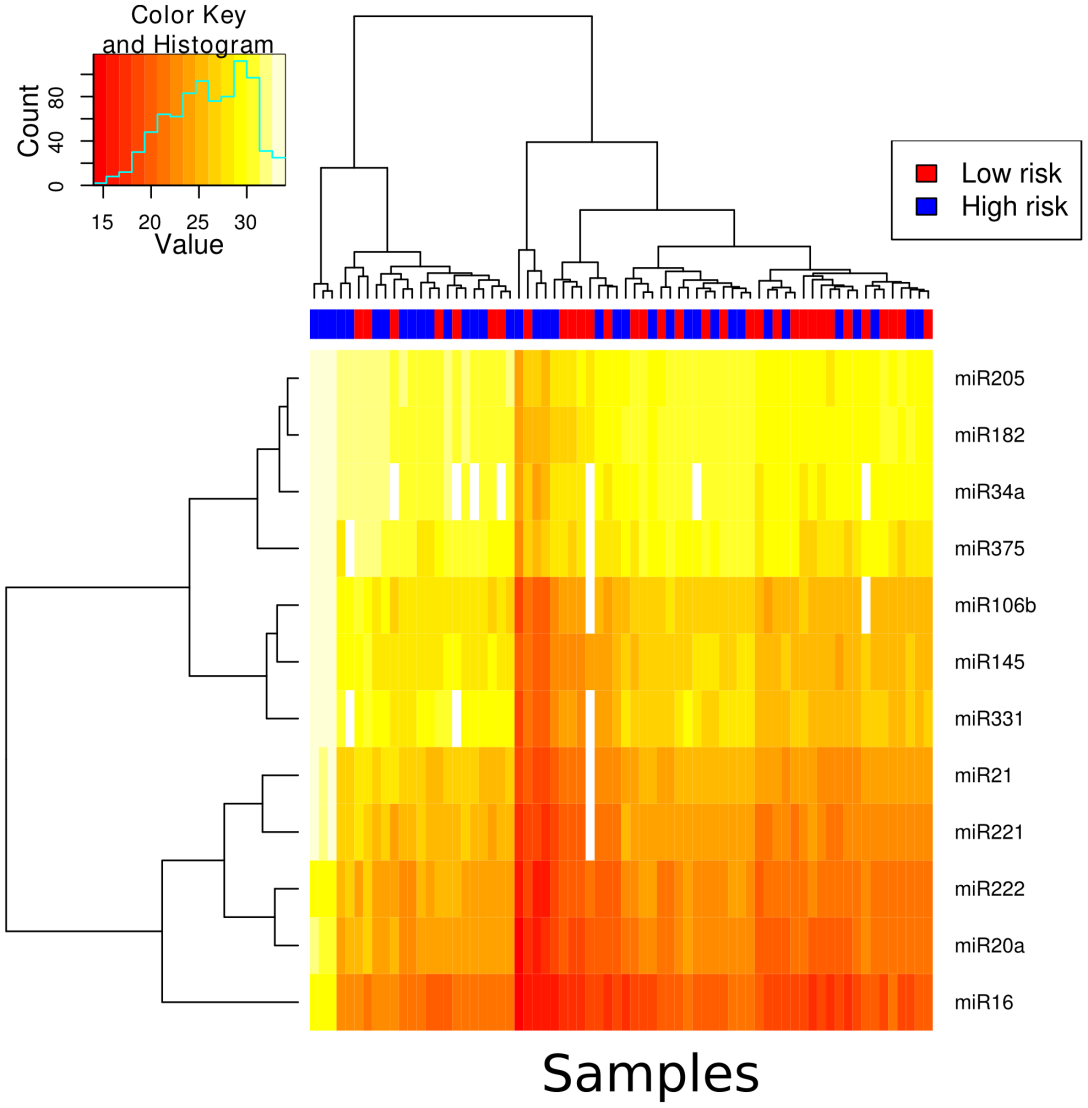
A heatmap showing the Ct values for each miR and each sample in the plasma cohort. The samples have been subjected to unsupervised hierarchical clustering and the results are depicted by the dendrogram at the top of the image.

**Figure 3:**
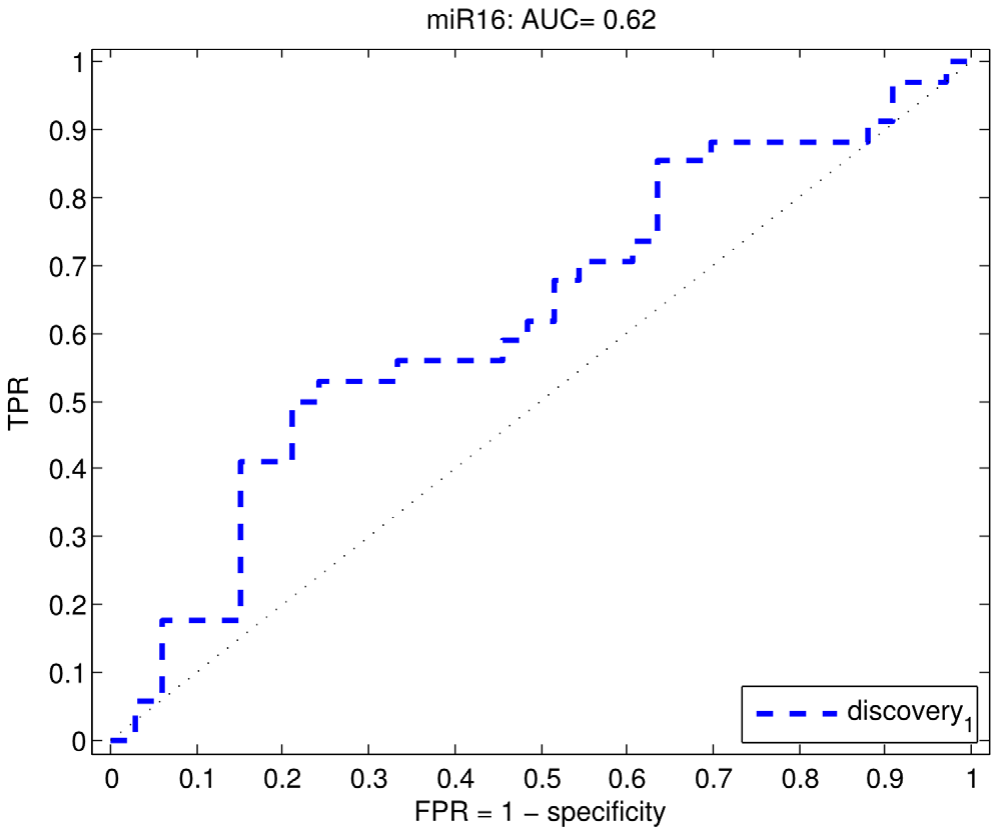
Receiver operating characteristic (ROC) curve for profiling of miRs in plasma. miR-16 does not accurately predict likelihood of developing high-risk disease at radical prostatectomy (AUC = 0.62).

The majority of miRNAs were at detectable concentrations in the plasma of patients with high-risk prostate cancer and low-risk prostate cancer (Figure 2). Three samples showed overall low detection rates and were removed from subsequent analysis. Unsupervised clustering was applied to samples across the 12 miRs profiled in plasma to determine if there was separation between high-risk and low-risk groups. There appeared to be no apparent separation between high and low-risk groups. We applied a feature selection procedure and tested the performance of the 12 miRs for their ability to delineate high-risk prostate cancer from low-risk prostate cancer. Table 2 shows the results from our feature selection procedure. Using both the t-test and centroid feature selection procedure, miR16 was selected as the most predictive feature, however, the ranking showed unstable results across all of the miRs. Figure 3, shows a ROC curve for miR16, demonstrating that the miR overall has quite poor performance with an area under the curve of 0.62. While predictive, we decided this performance was not sufficient to warrant further validation.

**Table 2:** This table contains the results of our feature selection procedure for miRs profiled in plasma.

Rank	Top 1	Top 2	Top 3	Top 4	Top 5	Top 6	Top 7	Top 8	miR
**T-test**									
**1**	33	37	47	53	60	67	70	70	miR16
**2**	13	37	47	53	57	57	57	63	miR145
**3**	17	40	40	50	53	60	67	73	miR106b
**4**	27	37	40	43	50	50	57	63	miR34a
**5**	3	10	27	33	37	43	57	70	miR182
**6**	0	0	13	30	43	50	57	63	miR20a
**7**	3	7	23	23	43	47	53	57	miR21
**8**	0	10	23	30	43	57	70	83	miR222
**9**	0	10	10	27	27	40	53	73	miR331
**10**	0	7	10	13	20	40	47	57	miR375
**11**	3	3	13	27	37	47	60	63	miR205
**12**	0	3	7	17	30	43	53	63	miR221
**Centroid**									
**1**	17	27	37	47	63	70	80	80	miR16
**2**	23	30	37	50	50	57	57	67	miR106b
**3**	13	33	57	63	70	70	73	73	miR145
**4**	13	30	33	37	40	47	60	60	miR34a
**5**	0	0	3	13	20	23	47	70	miR20a
**6**	7	10	10	20	23	40	43	60	miR182
**7**	0	7	30	37	40	50	63	80	miR21
**8**	0	3	10	23	37	47	47	57	miR222
**9**	13	33	40	53	60	63	73	73	miR331
**10**	3	7	10	13	27	43	53	63	miR375
**11**	10	20	30	37	53	63	70	77	miR221
**12**	0	0	3	7	17	27	33	40	miR205

The miRs have been ranked based on their appearance in the Top *n* miRs for different classifiers as part of the cross-validation procedure. The percentage of times the miR is selected by the classifier in the top n = 1..8 miRs is reported for both t-test and centroid approaches.

### microRNA profiling of urine

#### Discovery cohort

**Figure 4:**
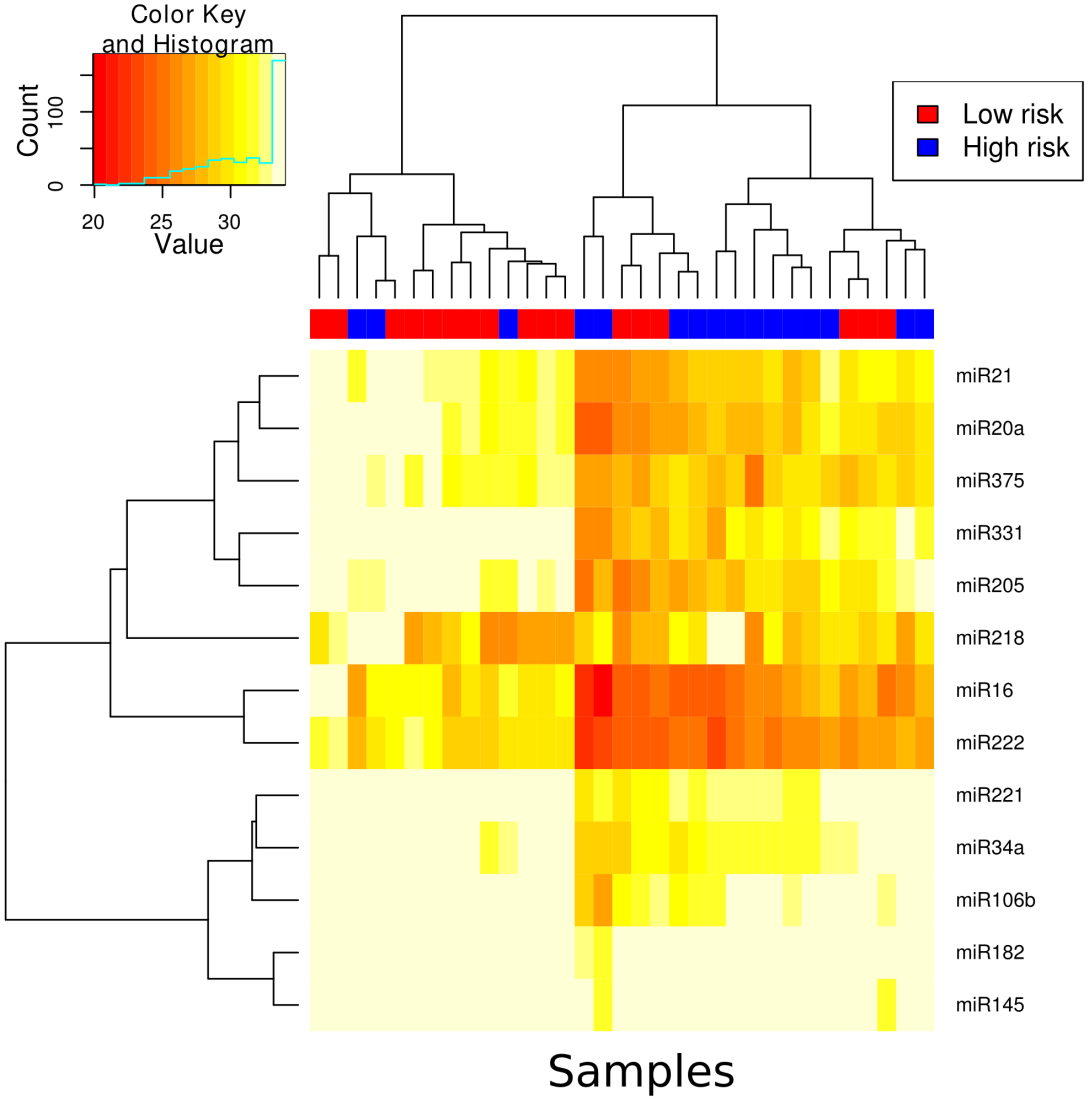
A heatmap showing the Ct values for each miR and each sample in the urine cohort. The samples have been subjected to unsupervised hierarchical clustering and the results are depicted by the dendrogram at the top of the image.

**Figure 5:**
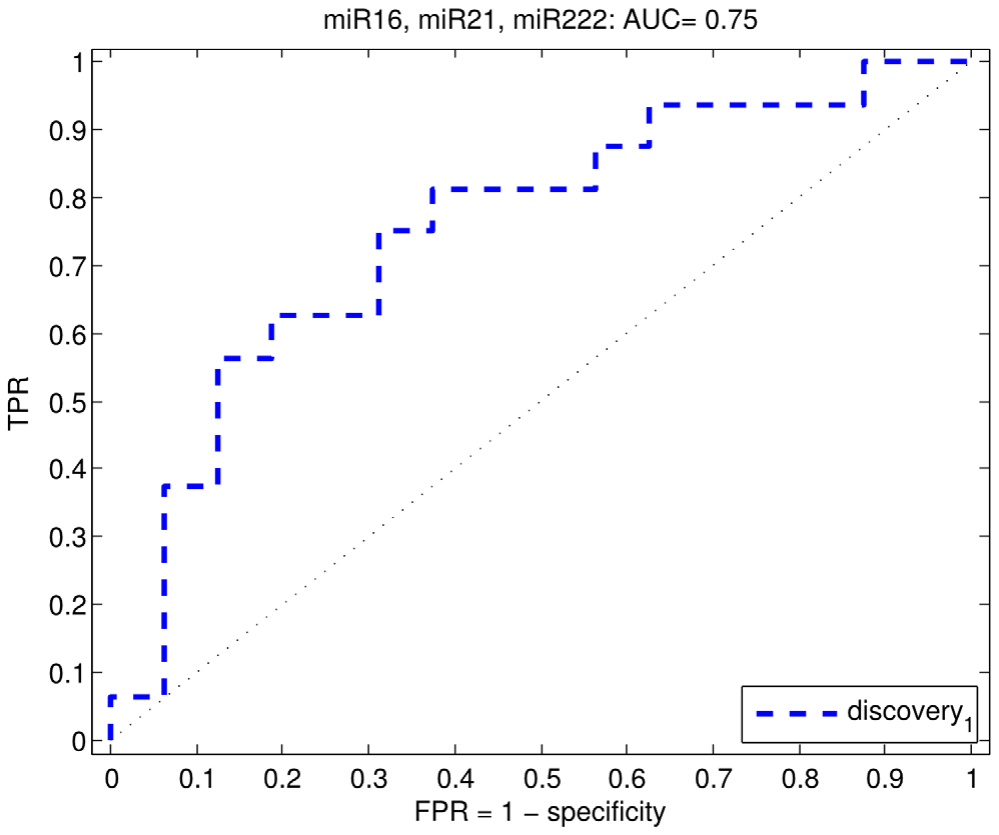
Receiver operating characteristic (ROC) curve for profiling of miRs in urine from prostate cancer patients. miR 16, miR 222 and miR 21 predict high-risk prostate cancer with an AUC of 0.75.

In screening urine, all samples showed a detectable level of expression for the selected miRs (Figure 4). Unsupervised clustering of the samples across all miRs showed a moderate separation of low-risk and high-risk samples (Figure 4). miRs 16, 20a, 21, 34a, 145, 106b, 182, 205, 221, 222, 331 and 375 were detected at higher levels in the high-risk group, whereas miR 218 was detected at lower levels in the urine of high risk prostate cancer patients. Of these miRs all but miR 218 were significantly different between the high and low risk groups (q-value<0.1) as shown in Table 3. The fold change for the miRNAs, which were significantly upregulated ranged from 2.17 (miR 145) to 8.09 (miR 222) as shown in Table 3. To determine the minimal set of miRs which showed the best separation of high and low risk groups, we used a feature selection procedure of which the results can be seen in Table 4. Both the t-test feature selection procedure and the centroid procedure selected miR16 and miR222 as the most predictive features. Beyond this, the ranking of miR performance was unstable. We therefore chose miR222 and miR16 for validation. In addition, we also chose miR21. Even though it did not rank highly on the feature selection procedure, we selected it as a feature that was likely to be independent from miR222 and miR16, as the least correlated miR within the cluster containing miR222 and miR16 (Figure 4). We tested the performance of all combinations of the 3 miRs and all three combined showed the best performance of AUC = 0.75 (Figure 5). We deemed this performance sufficient for further followup and sought a validation cohort.

**Table 3:** MicroRNA detection in urine of low-risk and high-risk prostate cancer patients at radical prostatectomy in the original cohort.

miR	Mean low-risk	Mean high-risk	ΔΔCt	Fold Change	P-value	Q-value
**miR 222**	22.6284	25.6460	3.017597	8.098176	0.013078	0.057554
**miR 16**	22.7880	25.8020	3.014036	8.078210	0.024273	0.057554
**miR 205**	25.8730	28.5610	2.687978	6.444098	0.038138	0.057554
**miR 20a**	25.2013	27.8736	2.672283	6.374369	0.042064	0.057554
**miR 331**	26.5414	29.1520	2.610608	6.107610	0.037799	0.057554
**miR 21**	25.6082	28.0528	2.444620	5.443823	0.039690	0.057554
**miR 375**	25.4324	27.8130	2.380519	5.207240	0.029020	0.057554
**miR 34a**	27.6115	29.8324	2.220869	4.661743	0.025066	0.057554
**miR 106b**	28.2883	30.1729	1.884607	3.692524	0.045221	0.057554
**miR 221**	28.4153	30.0484	1.633065	3.101714	0.060869	0.070955
**miR 182**	29.2585	30.6111	1.352615	2.553746	0.028318	0.057554
**miR 145**	29.3191	30.4363	1.117213	2.169275	0.065887	0.070955
**miR 218**	26.1297	25.6887	−0.441010	0.736619	0.612671	0.612671

**Table 4:** This table contains the results of our feature selection procedure for miRs profiled in urine.

Rank	Top 1	Top 2	Top 3	Top 4	Top 5	Top 6	Top 7	Top 8	miR
**T-test**									
**1**	60	90	97	100	100	100	100	100	miR222
**2**	7	33	50	60	77	77	80	83	miR16
**3**	7	23	37	60	67	90	90	93	miR34a
**4**	0	3	37	50	67	80	83	87	miR182
**5**	20	27	33	47	53	53	63	70	miR375
**6**	0	7	13	23	37	50	53	63	miR205
**7**	0	3	10	17	27	37	70	77	miR331
**8**	0	3	7	7	20	33	47	57	miR21
**9**	0	0	0	13	20	30	40	67	miR20a
**10**	0	0	3	7	10	23	33	43	miR106b
**11**	0	0	0	0	3	7	13	30	miR221
**12**	0	3	7	10	13	13	20	23	miR145
**Centroid**									
**1**	37	90	97	100	100	100	100	100	miR222
**2**	50	67	83	83	93	97	97	100	miR16
**3**	13	30	43	57	70	77	93	93	miR205
**4**	0	7	33	73	90	97	97	100	miR20a
**5**	0	3	27	50	80	90	97	100	miR331
**6**	0	3	10	10	20	63	93	100	miR21
**7**	0	0	7	17	23	43	70	87	miR375
**8**	0	0	0	3	10	20	40	93	miR34a
**9**	0	0	0	0	3	3	3	13	miR106b
**10**	0	0	0	7	10	10	10	13	miR218
**11**	37	90	97	100	100	100	100	100	miR222
**12**	50	67	83	83	93	97	97	100	miR16

The miRs have been ranked based on their appearance in the Top *n* miRs for different classifiers as part of the cross-validation procedure. The percentage of times the miR is selected by the classifier in the top n = 1..8 miRs is reported for both t-test and centroid approaches.

#### Validation cohort

The demographics and clinicopathological characteristics of the low-risk and high-risk patients in cohort 2 (validation cohort) are outlined in Table 5. Validation was only undertaken for urinary expression as the classifier for plasma expression of miRNA yielded only moderate performance.

**Table 5:** Clinical and pathological characteristics of patients in the validation cohort.

		Urine cohort	
		Low-Risk	High-Risk
n		14	22
Age	Median	63	63
(yrs)	Range	45–71	49–79
Follow-up	Median	3.47	3.84
(yrs)	Range	2.79–5.13	2.77–5.41
Prostate Weight	Median	60	50
(g)	Range	28–78	31–87
Gleason Grade (%)	6	14 (100)	0 (0)
	7 (3+4)	0 (0)	0 (0)
	7 (4+3)	0 (0)	1 (4)
	8	0 (0)	3 (14)
	9 (4+5)	0 (0)	15 (68)
	9 (5+4)	0 (0)	3 (14)
Tumour Volume	Median	.2000	10.65
(cc)	Range	0.10–0.90	2.50–28.70
pT stage (%)	pT2a	4 (29)	0 (0)
	pT2b	0 (0)	0 (0)
	pT2c	10 (71)	1 (5)
	pT3a	0 (0)	10 (45)
	pT3b	0 (0)	11 (50)
EPE (%)	Present	0 (0)	21 (96)
	Absent	14 (100)	1 (4)
SV Invasion (%)	Present	0 (0)	10 (45)
	Absent	14 (100)	12 (55)
PN Invasion (%)	Present	3 (21)	22 (100)
	Absent	11 (79)	0 (0)
LV Invasion (%)	Present	0 (0)	16 (73)
	Absent	14 (100)	6 (27)
Multifocal (%)	Yes	12 (86)	13 (59)
	No	2 (14)	9 (41)
Surgical Margins (%)	Positive	0 (0)	16 (73)
	Negative	14 (100)	6 (27)
Metastatic Disease (%)	Yes	0 (0)	0 (0)
	No	14 (100)	22 (100)

**Figure 6:**
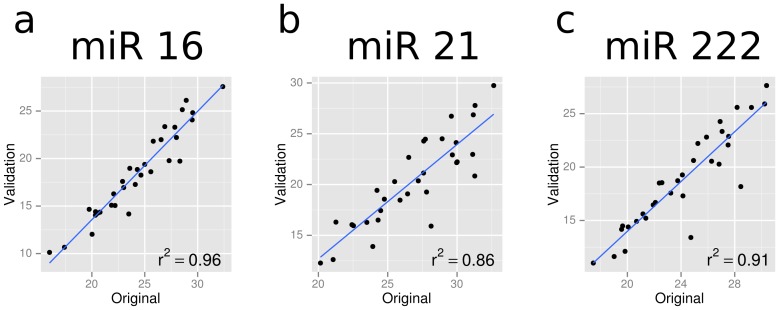
Ct values of three miRs profiled across the same samples using two independent platforms. R^2^ values represent Pearson correlation.

**Figure 7:**
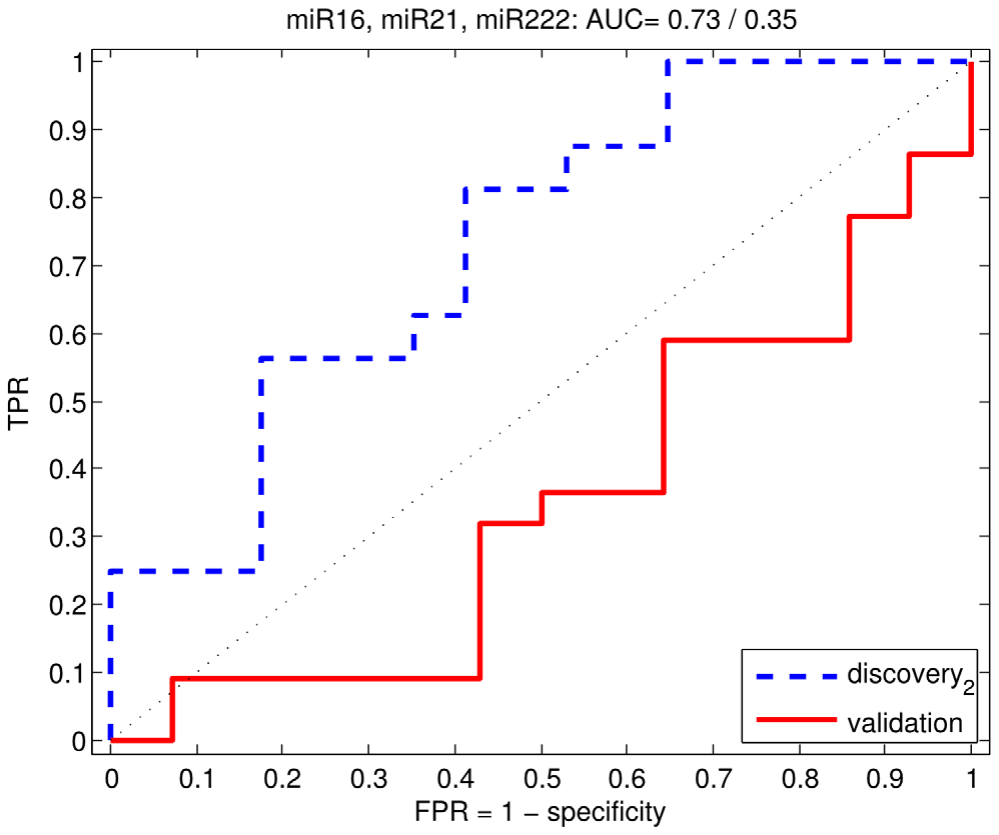
Receiver operating characteristic (ROC) curve for profiling of miRs in urine. Prostate cancer patients from the original cohort (blue). Prostate cancer patients from the validation cohort (red).

We chose miR 16, 21 and 222 for validation in an independent cohort of low-risk and high-risk prostate cancers. We chose a different method of profiling of miRNAs in the validation cohort to test for robustness, as described in the methods. To test for uniformity of detection across the two platforms, we profiled the samples from the original cohort on the new platform and observed the correlation of Ct values between the platforms for the three miRs. All three miRs showed high correlation between platforms (Figure 6) suggesting that detection of these miRs was robust. Their performance as predictors of high-risk disease was also comparable across platforms (Figure 7, AUC = 0.72). However, as shown in Table 6, in the validation cohort, we found that the three miRNAs were surprisingly detected at lower levels in the high-risk group, compared to the low-risk group: miR 16 (FC = 0.07, q = 0.08), miR 21 (FC = 0.14, q = 0.10) and miR 222 (FC = 0.14, p = 0.10). When the classifier built on the original cohort using the three miRs was used on the validation cohort, we achieved a performance of AUC = 0.35 (Figure 7).

**Table 6:** MicroRNA expression in urine of low-risk and high-risk prostate cancer patients at radical prostatectomy in the validation cohort.

miR	Mean low-risk	Mean high-risk	ΔΔCt	Fold Change	P-value	Q-value
**miR21**	23.51441	20.68556	−2.82885	0.14074	0.09184	0.09614
**miR222**	22.35019	19.48983	−2.86037	0.13770	0.09614	0.09614
**mir16**	23.22368	19.35065	−3.87303	0.06825	0.02804	0.08412

#### Evaluation of methodology of miRNA profiling

To evaluate if this change in expression pattern of the miRNAs was due to methodological difference, we profiled for the three miRNAs using both methodologies on the original discovery cohort. We found that using both methodologies, all three miRNAs were detected at higher levels in the high-risk group in the original discovery cohort. Comparison of expression of these three miRNAs using both methodologies on the original cohort showed good correlation (Correlation coefficient R^2^ = 0.96 (miR 16), R^2^ = 0.91 (miR 222) and R^2^ = 0.86 (miR 21)) confirming that the difference observed was due to a biological variation rather than methodological variation.

## Discussion

Predicting high-risk CaP remains a challenge and this underpins our current overtreatment of biologically insignificant cancers. For any biomarker to be successfully translated to the clinical arena, it must withstand sufficient validation in independent cohorts of patients and demonstrate methodological robustness. In this study we have highlighted the importance of validating results in an independent cohort using alternate methodological platforms. In the original cohort, we found that miR 16, 222 and 21 were upregulated in high risk prostate cancer compared to low-risk cancer and urinary profiling of miR 16, miR 222 and miR 21, was able to distinguish between those cancers likely to have an indolent course from the biologically significant cancers after radical prostatectomy. However these same miRNAs were found to be detected at lower levels in an independent cohort of high-risk prostate cancers compared to low-risk cancers. This is in contrast to the results from the original cohort. Thus profiling for these miRNAs in plasma or urine, in our study, did not robustly distinguish between low-risk and high-risk CaP.

Given that miRNA profiling using both these distinct methodologies on the original discovery cohort yielded similar results, this is most likely to represent genuine biological variation rather than a difference due to methodological variation. The TLDA cards are a relatively new platform for performing PCR that haven't been subjected to intense validation but have been used by many groups previously for quantifying gene expression [23], [24], [25]. Profiling of miR 16, 21 and 222 on the samples in the original cohort using the two different methodologies showed that the results correlated well. This confirmed that the experimental methodology was robust and results are indicative of a true biological variation in the expression of these miRNAs in the two independent groups.

The analysis of urine as a diagnostic or prognostic tool is not unprecedented. Levels of protein, mRNA, miRNA and even gene methylation in urine have been correlated with cancer presence and aggression in prior studies [26], [27], [28], [29], [30]. In addition, others have found urine to be a better source of prostate material than plasma [28]. The method of these cellular substances reaching the urine is likely via transport of cancer cells through the prostatic ductal system [30] or the exosomal pathway [27].

Many previous studies have studied the regulation of miRNA species in prostate cancer tissues relative to benign prostate, high-grade primary relative to low-grade, or metastatic relative to primary cancer [31], [32], [33]. Additionally, many of the miRNAs involved in prostate cancer initiation, progression and metastasis have been functionally characterised [34], [35]. Recent work also profiled miRNA species found to be upregulated or downregulated in plasma or serum of prostate cancer patients [10], [11], [12], [13], [36], [37]. These studies have revealed that specific miRNAs may be useful for a non-invasive prognostic test, but only using plasma or serum. Few studies have profiled for miRNAs in urine from patients with prostate cancer. They have found that several circulating miRNAs are differentially expressed in prostate cancer [10], [11], [12], [13], [36], [37], [38]. Some have found that miR 26a, 30c and let 7 are differentially expressed in plasma or serum of patients with prostate cancer compared to those with benign prostatic hyperplasia (BPH)[12], [36]. Others have compared patients with low-risk to high-risk prostate cancer and across various studies miR-141, miR-375, miR-221, miR-21 and miR-145 have shown to be elevated in patients with high-risk or metastatic prostate cancer [10], [11], [37], [38]. Whilst miR 222 has not been shown to be significantly predictive of high-risk disease in previous studies [39], miR 21 has been shown to be elevated in more aggressive prostate cancer in some studies [11], [37]. Shen et al found that miR 21 levels correlated with Cancer of the Prostate Risk Assessment (CAPRA) scores [11] and Agaoglu and colleagues found that miR 21 levels were elevated in patients with metastatic tumors compared to those with localized disease [37]. Similarly miR 221 has been shown to be elevated in both these studies but miR 221 was not consistently elevated in high-risk tumors in our cohort.

However these studies have repeatedly yielded different miRNA signatures to risk stratify prostate cancer due to varying experimental methodology, lack of robustness of statistical analysis and non-standardised definitions of high-risk or low-risk prostate cancer. We have tried to address some of these problems by using robust statistical approaches for analysis of results as well as using standardised definitions of indolent (Epstein criteria) and high-risk disease. The majority of the previous studies have not included validation of significant miRNAs and hence they lack the ability to demonstrate reproducibility of data [11], [12], [13], [36], [37].

Another point to be highlighted is that the samples in other studies done so far were taken from patients when they had developed metastatic disease. Whilst this provides insight into the biology of tumour progression, it doesn't allow risk stratification of patients when they still have localised disease and as such doesn't have an immediate clinical implication. Our study is novel in that all plasma and urine samples were collected prior to radical prostatectomy and hence at the time when risk stratification, selection for treatment and prognostication of patients is paramount.

Other tests commercially available for risk stratification of prostate cancer are the PCA3 and the PHI test. The PCA3 test, which is a urine-based mRNA test, is more specific for prostate cancer than the PSA test [30], [40]. It has been shown to be useful in guiding decisions for prostate biopsy based on a cumulative score calculated by measuring the expression of the prostate cancer gene mRNA. More recently it has been shown to be predictive of ‘significant’ prostate cancer as there was a significant difference in PCA3 scores of patients who satisfied the Epstein criteria for indolent cancers [41] and ‘significant’ cancers that were deemed appropriate for radical prostatectomy. The PHI test which measures a different isoform of PSA, the [−2] pro-PSA, has been found to be useful in predicting prostate cancer with Gleason score≥7 in patients with PSA of 2–10 ng/ml however is not associated with prostate volume [42]. However, neither of these tests help to determine those people likely to develop metastatic disease after radical prostatectomy.

There are some limitations of this study that need to be enumerated. With no accepted biological control for miRNAs in biofluids, we haven't normalised our results to a ‘house-keeping’ gene. Several endogenous controls are used in tissue profiling studies. However when we used RNU48 as an endogenous control, we found that its expression was not consistent in the low-risk and high-risk groups. With such a systematic perturbation in its expression, it was unsuitable as an endogenous control. With time, as miRNAs are studied further in biofluids, certain biological controls might emerge. Secondly urine is a dynamic body fluid and concentrations can change with hydration status and renal pathology. In our study all urine samples were collected immediately prior to radical prostatectomy hence there is likely to be a high degree of uniformity within the urine compositions. Measuring 24-hour urine volumes would be the gold standard to assess hydration status but this is laborious, expensive and fraught with low compliance rates in the clinical setting. Measuring creatinine ratios or specific gravity remain other possibilities and potentially more feasible alternatives. Finally our sample size is small.

To conclude, here we have shown plasma and urinary profiling of miRNAs may not be a robust marker for prognostication of prostate cancer aggression and high-risk disease. This study highlights the importance of incorporating validation cohorts and methodological robustness to ensure reproducibility of data in all future biomarker studies.
